# Dr. Livingston

**Published:** 1857-04

**Authors:** 


### *Dr. Livingston.*

Dr. Livingston, who lately returned to Great Britain, from an absence of nearly seventeen years in the wilds of South Africa, is undergoing the ordeal of a degree of public attention, quite unusual with our phlegmatic neighbors. But he well deserves it and tenfold more. As an example of a persevering and self-sacrificing man of science and a zealous missionary, he has probably no equal in the ranks of the Christian Church, or in the medical profession. In addition to all, his modesty in positively refusing to publish to the world the details of the dangers he has encountered, is equally rare, and although carried to an unnecessaty extreme, forms an agreeable contrast to the romantic accounts of some professedly scientific travellers, whom we might name. In one of his public addresses, he, however, mentioned incidentally that for *sixteen years* he had not an opportunity of conversing in his native language, a circumstance which is of itself significant to our minds of positive suffering seldom endured, even within the walls of a state prison. His contributions to scientific knowledge have not yet been given to the public, but doubtless will be ere long, when we look for some most interesting additions to our present slender information, concerning the diseases of the countries through which he traveled. In his address before the London Missionary Society, he stated that some of the interior districts of Africa are perfect sanatoria, and among the pure negro family, many diseases common in Europe are almost unknown. Small-pox and measles had not been known for twenty years, and consumption, scrofula, cancer, and hydrophobia are seldom heard of.—*North American Med. Chirurg. Review.*

